# Synthesis, Production, and Biotechnological Applications of Exopolysaccharides and Polyhydroxyalkanoates by Archaea

**DOI:** 10.1155/2011/693253

**Published:** 2011-10-10

**Authors:** Annarita Poli, Paola Di Donato, Gennaro Roberto Abbamondi, Barbara Nicolaus

**Affiliations:** ^1^Institute of Biomolecular Chemistry (ICB), National Research Council (CNR), Via Campi Flegrei 34, 80078 Pozzuoli, Italy; ^2^Department of Environmental Sciences, University of Naples “Parthenope”, Centro Direzionale, Isola C4, 80143 Naples, Italy

## Abstract

Extreme environments, generally characterized by atypical temperatures, pH, pressure, salinity, toxicity, and radiation levels, are inhabited by various microorganisms specifically adapted to these particular conditions, called extremophiles. Among these, the microorganisms belonging to the Archaea domain are of significant biotechnological importance as their biopolymers possess unique properties that offer insights into their biology and evolution. Particular attention has been devoted to two main types of biopolymers produced by such peculiar microorganisms, that is, the extracellular polysaccharides (EPSs), considered as a protection against desiccation and predation, and the endocellular polyhydroxyalkanoates (PHAs) that provide an internal reserve of carbon and energy. Here, we report the composition, biosynthesis, and production of EPSs and PHAs by different archaeal species.

## 1. Introduction

A vast number of EPSs from extremophiles were reported over the last decades, and their greatly variable composition, structure, biosynthesis and functional properties have been extensively studied but only a few of them have been industrially developed. EPSs are highly heterogeneous polymers containing a number of distinct monosaccharides and noncarbohydrate substituents that are species specific. Polysaccharide chains are usually formed by using an oligosaccharide as a repeating unit that can vary in size depending on the degree of polymerization. Exopolysaccharides have found multifarious applications in the food, pharmaceutical, and other industries. Both extremophilic microorganisms and their EPSs suggest several biotechnological advantages, like short fermentation processes for thermophiles and easily formed and stable emulsions of EPSs from psychrophiles [1–4].

EPSs have been isolated from different genera of Archaea, mainly belonging to thermophilic and halophilic groups. Thermophilic (heat loving) microorganisms can be found in every phylum of Archaea and Bacteria, and have been isolated from various thermophilic ecosystems: marine hot springs, both deep and shallow, and terrestrial hot springs that have served as sources for isolation of microbial EPS producers. Among the thermophilic archaeal genera, *Thermococcus *and *Sulfolobus* produce EPSs, and *Archaeoglobus fulgidus *and *Thermococcus litoralis* accumulate significant amounts of EPSs as biofilms [5–8], a consortium of microorganisms immobilized and penned within EPS, which can restrict the diffusion of substances and antimicrobial agents.

Beside archaea, several thermophilic bacteria are good producers of large amounts of EPS such as *Bacillus thermantarcticus*,* Geobacillus thermodenitrificans*, and *Bacillus licheniformis*, isolated from hot marine shallow vents, or as extremely thermophilic fermentative anaerobe *Thermotoga maritima* and cocultures of *Thermotoga maritima *and the H_2_-consuming methanogen *Methanococcus jannaschii*, that were found to develop significant biofilms, or finally as *Geobacillus tepidamans *V264, isolated from a terrestrial hot spring, that is able to produce an unusually thermostable exopolysaccharide, that starts to decompose at about 280°C [9]. Although many thermophiles have been isolated from hot springs, there are few studies of their biofilm EPS. Lin et al. [10] characterized the primary structure of a novel exopolysaccharide TA-1 secreted by *Thermus aquaticus *YT-1 showing that TA-1 possesses immunological activity. *T. aquaticus *and other thermophiles may be protected from environmental stress, such as high temperature, by biofilm, unusual glycolipids, and high DNA GC contents [10].

Many halophilic Archaea were established as being EPS producers such as *Haloferax*, *Haloarcula*, *Halococcus*, *Natronococcus*, and *Halobacterium *[11–14]. Nevertheless, the most common halophilic EPS producers are bacteria belonging to the genus *Halomonas*, most importantly *H. maura, H. eurihalina, H. ventosae,* and *H. anticariensis *[15]. Exopolysaccharides synthesized by *Halomonas *strains had an unusually high sulphate content and a significant amount of uronic acids determining their good gelifying properties. Other good halophilic EPS producers belonging to the gamma-proteobacteria were the genera *Idiomarina* and *Alteromonas*, namely *Alteromonas hispanica*; moreover, the alpha-proteobacteria *Salipiger mucosus *and *Palleronia marisminoris* were reported as EPS producers [15, 16].

The endocellular polyhydroxyalkanoates (PHAs), polyesters composed of hydroxy fatty acids, are biosynthesized and stored as lipid inclusions: it is generally accepted that microorganisms isolated from a natural environment poor in nutrient sources (from soil or spring water) exhibit a higher survival ability than those living in the alimentary tract of higher organisms [17]. Indeed several extreme microorganisms accumulate these lipid inclusions as they enter the stationary phase of growth, to use them later as an internal reserve of carbon and energy [18, 19]. 

PHAs are produced by some halophilic archaeal species belonging to the genera *Haloferax*, *Haloarcula*, *Natrialba*, *Haloterrigena*, *Halococcus*, *Haloquadratum, Halorubrum*, *Natronobacterium*, *Natronococcus, *and *Halobacterium *[12, 20–29]. Moreover, PHAs are produced also by several bacteria, some of which has been isolated from the waste stream of several treatment facilities that often provide a mixture of substrates [30]. PHA production have been reported among diverse bacteria, including *Bacillus*, which were widely studied since the discovery of poly-*β*-hydroxybutyrate (PHB) in a *B. megaterium* strain in 1925. PHB, the best known polyhydroxyalkanoate, has also been reported in *Methylobacterium, Staphylococcus, Micrococcus*, and *Rhodococcus* and *Paracoccus*. Strains of *Methylobacterium, Paracoccus*, and *Rhodococcus *could be candidates for producing copolymers, such as poly ([R]-3-hydroxybutyrate-co-hydroxyvalerate) (PHBV), under limited nitrogen conditions using acetate, glucose, methanol, pentothal, propionic acid, or valeric acid as a substrate [12, 30, 31]. 

This paper represents a short review of archaeal microorganisms as a source of EPSs and PHAs, with particular attention to their production and potential biotechnological applications.

## 2. EPSs: Characteristics and Physiological Roles

Exopolysaccharides (EPSs) are high-molecular-weight polymers that are composed of sugar residues and are secreted by microorganisms into the surrounding environment. They make up a substantial component of the extracellular polymers surrounding most microbial cells in extreme environments like Antarctic ecosystems, saline lakes, geothermal springs, or deep sea hydrothermal vents. The extremophiles have developed various adaptation strategies, enabling them to compensate for the deleterious effects of extreme conditions, high temperatures and salt concentrations, low pH or temperature, and high radiation. Among these strategies, the EPS biosynthesis is one of the most common protective mechanisms. In their natural environment, most bacteria occur in microbial aggregates whose structural and functional integrity is based on the presence of a matrix of extracellular polymeric substances and the EPS production seems to be essential for their survival [32]. Many microorganisms (many species of Gram-positive and Gram-negative bacteria, archaea, fungi and some alga) are known to produce extracellular polysaccharides. Exopolysaccharide is a term first used by Sutherland [33] to describe high-molecular-weight carbohydrate polymers produced by marine bacteria. Exopolysaccharides can be found as in capsular material or as dispersed slime in the surrounding environment with no obvious association to any one particular cell [34]. Considerable progress has been made in discovering and developing new microbial EPSs that possess novel industrial significance [15]. A vast number of microbial EPSs were reported over the last decades, and their composition, structure, biosynthesis and functional properties have been extensively studied. In recent years the increased demand for natural polymers for pharmaceutical, food, and other industrial applications has led to a remarkable interest in polysaccharides produced by microorganisms. Indeed, a substantial interest has aroused with regard to the isolation and identification of new microbial polysaccharides that might have innovative applications as gelling, emulsifier and stabilizer agents [35].

Many microorganisms produce exopolysaccharides as a strategy for growing, adhering to solid surfaces, and surviving adverse conditions. The physiological role of EPS depends on the ecological niches and the natural environment in which microorganisms have been isolated. Indeed, the EPS production is a process that requires a noticeable energy cost of up to 70% of total energy reserve, representing a significant carbon investment for microorganisms. However, the benefits related to EPSs production are significantly higher than costs considering the increasing growth and survival of microorganisms in their presence [36]. Indubitably, EPSs possess a protective nature: they form a layer surrounding cells provide an effective protection against high or low temperature and salinity or against possible predators. They are essential in the aggregate formation, in the mechanism of adhesion to surfaces and to other organisms, in the formation of biofilm and in the uptake of nutrients [2, 37]. In particular, studies of sea ice microbial communities have also found bacteria strongly associated to particles and have pointed out that microbial EPSs played an important role in cryoprotection [38].

## 3. EPSs: Chemical Studies and Compositions

An overall characterization of biopolymers involves the evaluation of their chemical, physical, and biological properties, being a key factor in order to understand their behavior in different environments, which enables to foresee their potential applications. Chemical characterization concerns the identification of sugar residues, repeating units (which may be formed by more than one sugar/sugar based molecule), and chain groups constituents (e.g., acyl and phosphate groups). The traditional method, consisting of acid hydrolysis followed by derivatisation to alditol acetates assayed by gas chromatography, has been gradually replaced by high pressure anion exchange chromatography with pulsed amperometric detection (HP-AEC-PAD), which is more straightforward avoiding the derivatisation step. Characterization of single carbohydrates using capillary electrophoresis (CE), without significant carbohydrate modification after hydrolysis, has been also well documented. The possible multiple combinations of monomeric units, along with the stereospecificity of glycosidic linkages (*α*- or *β*- anomers), lead to very complex chemical structures quite difficult to resolve, ranging from linear homopolysaccharides to highly branched heteropolysaccharides. The linkage pattern of the monomers is evaluated by methylation: according to the most used method, all free hydroxyl groups undergo methylation, followed by polysaccharide hydrolysis, reduction of methyl glycosides by NaBD_4_ and acetylation, which provides *O*-acetyl group at linkage points. The partially methylated alditol acetate is analysed by GC-MS [31, 41]. This study has been complemented with improved liquid state 2D NMR methodologies (such as COSY, NOESY, TOCSY and HSQC), which allow to show the environment where each carbon/hydrogen is positioned as well as gel and solid state NMR [14, 42, 43]. Various techniques have been used for the determination of polymer molecular mass such as high performance size exclusion chromatography with multi-angle laser light scatter detection (HP-SEC-MALLS) [44], a recent efficient method for the evaluation of polysaccharide absolute molecular mass, that provides greater resolution than traditional gel permeation chromatography (GPC).

Sletmoen et al. [43] reported recent advancements in the studies of polysaccharides at the single-molecule level. Over the last few years, single-molecule techniques such as fibre diffraction, transmission electron microscopy (TEM), and the atomic force microscopy (AFM), have improved in sensitivity giving a good opportunity to investigate properties of single molecules close to physiological conditions. While X-ray diffraction can be used for structure determination on the atomic scale for establishing the three-dimensional structure and organization of long chain polymers, imaging by the ultramicroscopic techniques TEM and AFM provides information in the nm to *μ*m range. These techniques can be used to determine, for example, the distribution of the polymer chain lengths, polymer chain flexibility, and the mass per unit length. However, it has to be underlined that TEM requires elaborated preparation procedures to achieve contrast enhancement and vacuum compatibility of sample; therefore the presence of possible artifacts related with the preparation procedure needs proper attention in order to avoid possible misinterpretations of the image. On the contrary, AFM has the advantage of offering an operating environment close to physiological conditions, with the images being recorded in air or when the sample is immersed in liquid, such as water [43]. 

Most EPSs are heteropolysaccharides containing three or four different monosaccharides arranged in groups of 10 or less to form the repeating units. These polymers are often linear with an average molecular weight ranging from 1 × 10^5^ to 3 × 10^5^ Da. They are generally constituted by monosaccharides and noncarbohydrate substituents (such as acetate, pyruvate, succinate, and phosphate). Some EPSs are neutral macromolecules, but the majority of them are polyanionic for the presence of uronic acids or ketal-linked pyruvate or inorganic residues. The EPSs synthesized by microbial cells vary greatly in their composition and hence in their chemical and physical properties. Components most commonly found in EPS are monosaccharide such as pentoses (as D-arabinose, D-Ribose, and D-Xylose), hexoses (D-Glucose, D-Galactose, D-Mannose, D-Allose, L-Rhamnose, L-Fucose), amino sugars (D-Glucosamine and D-Galactosamine) or uronic acids (D-Glucuronic acids and D-Galacturonic acids). Organic or inorganic substituents such as sulphate, phosphate, acetic acid, succinic acid and pyruvic acid may also be present. The linkages between monosaccharides that have been most commonly found are 1,4-*β*- or 1,3-*β*-linkages in the backbones characterized by strong rigidity and 1,2-*α*- or 1,6-*α*-linkages in the more flexible ones. The physical properties of polysaccharides are deeply influenced by the way the monosaccharides are arranged together and by the assemblage of the single polymer chains [45]. The composition and structure of the polysaccharides determine their primary conformation. Furthermore, the ordered secondary configuration frequently takes the form of aggregated helices. The transition in solution from random coil to ordered helical aggregates is often greatly influenced by the presence or absence of acyl substituents such as *O*-acetyl or *O*-succinyl esters or pyruvate ketals [45].

## 4. Archaeal EPS Producers

Exopolysaccharides were identified in different groups of Archaea, predominantly in halophiles and thermophiles (Table 1). Massive amounts of EPSs are excreted by members of the halophilic genera *Haloferax*, *Haloarcula*, *Halococcus*, *Natronococcus, *and *Halobacterium *[11–14, 39]. Various thermoacidophilic archaea, including members of the genera *Thermococcus *and *Sulfolobus*, were observed to accumulate storage polysaccharides, such as glycogen, and to secrete mannan and sulphated heteropolysaccharide, respectively [5, 7]. *Archaeoglobus fulgidus *and *Thermococcus litoralis *accumulated significant amounts of EPSs as biofilms [6–8].

Antón et al. [11] were the first that reported the production of EPS by an archaebacterium. The authors described *Haloferax mediterranei* (ATCC 33500) as a producer of an exocellular polymeric substance that gave a typical mucous character to the colonies and was responsible for the appearance of a superficial layer in unshaken liquid medium. They obtained the EPS from the supernatant of shaken liquid cultures by cold ethanol precipitation with a yield as high as 3 mg/mL using glucose as carbon source. The polymer was identified as a heteropolysaccharide containing mannose as the major component. Glucose, galactose, and another unidentified sugar were also detected, as well as amino sugars, uronic acids, and a considerable amount of sulfate, which accounts for the acidic nature of the polymer.

The structure of the repeating unit of this polymer was subsequently determined by Parolis et al. [14] by a combination of glycose, methylation, and sulfate analysis, periodate oxidation, and 1D and 2D NMR spectroscopic analysis of the native and periodate-oxidised/reduced polysaccharides as →4)-*β*-D-Glc*p*NAcA-(1→6)-*α*-D-Man*p*-(1→4)-*β*-D-Glc*p*NAcA-3-O-SO_3_^−^-(1→. As expected, the elucidation of the chemical structure confirmed that this polymer is a highly charged molecule as previously described [11].

The structure of the neutral exocellular polysaccharide isolated from *Haloferax gibbonsii* (ATCC 33959) has been determined by Paramonov et al. [13]. The polysaccharide contained D-Man, D-Glc, D-Gal and L-Rha in the ratios of 2 : 1 : 3 : 1. The substitution patterns of the sugar residues were deduced from the methylation analysis which indicated a heptasaccharide repeating unit containing two branches. The sequence of sugars in the repeating unit (Table 1) was determined by means of NOESY and HMBC NMR experiments. 

Parolis et al. [39] reported the structure of a linear, acidic exopolysaccharide isolated from *Haloferax denitrificans*, an extremely halophilic organism which grows in the presence of salt concentrations ranging from 1.5 to 4.5 M. This archaeon is aerobic, highly pleomorphic and produces orange-red colonies. The sugar residues in the repeating unit of the polysaccharide were identified as →4)-*β*-D-Glc*p*A2,3NAc-(1→4)-*β*-D-Glc*p*A2,3NAc-(1→4)-*α*-D-Glc*p*A2,3NAc-(1→3)-*α*-D-Gal*p*-(1→, where D-Glc*p*A2,3NAc is 2,3-diacetamido-2,3-dideoxy-D-glucopyranosiduronic acid. 

**Figure 1:**
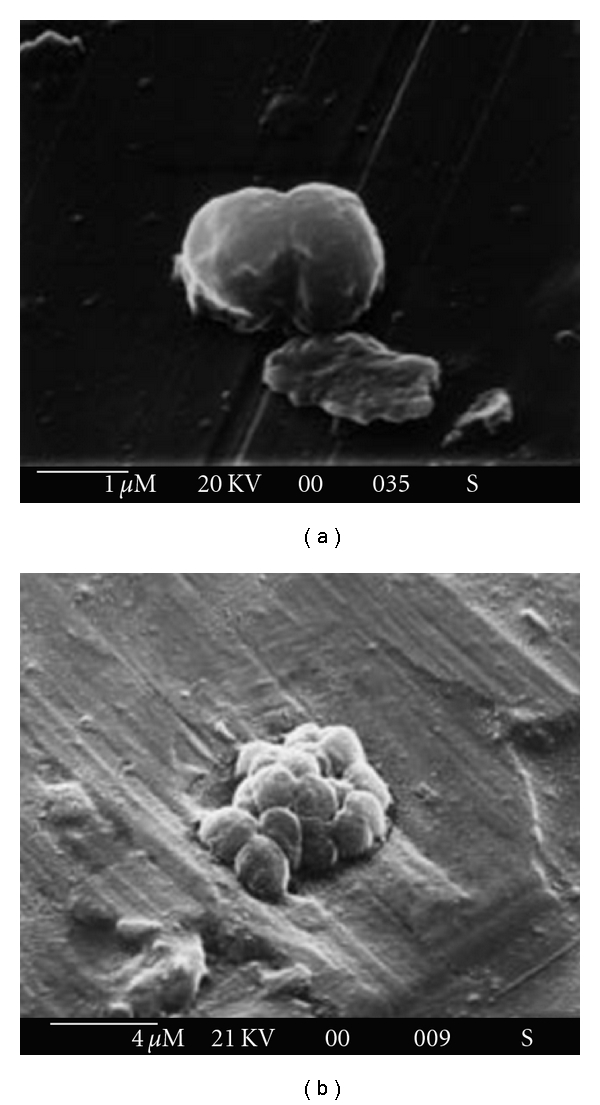
Scanning electron microscope (SEM) imagine of *Haloarcula japonica* strain T5 (DSM 12772). (a) Cells grown on standard complex medium. (b) Biofilm production of strain T5 grown under standard conditions with 160 mM Mg^+2^. The bar indicates the dimensions. By Nicolaus et al. [12].

In a screening program to obtain new polyhydroxyalkanoate and exopolysaccharide producers, Nicolaus et al. [12] isolated three obligately halophilic microorganisms (named T5, T6, and T7 strains) from an unexplored site in Tunisia (Monastir). All the isolates had polar lipid patterns characteristic of the representatives of the genus *Haloarcula* and in particular T5 strain was identified as a new strain of *H. japonica* by the DNA—DNA hybridization analysis (Figure 1) [12]. These strains were grown on a minimal medium containing glucose as sole carbon source: in such conditions they showed to be able to produce sulfated extracellular polysaccharides, that were easily isolated from cell free culture broth by precipitation with cold ethanol. The EPS yields obtained were 370 mg/L, 45 mg/L and 35 mg/L for T5, T6 and T7 strains, respectively. Sugar analysis of EPS from strain T5 revealed as principal constituents mannose, galactose, and glucuronic acid in a relative proportion of 2 : 1 : 3, respectively. Moreover, sugar analysis of crude EPSs of strains T6 and T7 yielded, as principal constituents mannose, galactose and glucose in the same relative proportion of 1 : 0.2 : 0.2.

The extreme thermoacidophile *Sulfolobus solfataricus *strain MT4 and strain MT3 were observed to produce a sulfated-, glucose-, mannose-, glucosamine-, and galactose-containing exopolysaccharide during growth under optimal conditions. The maximum production was reached during the stationary phase of growth and the yields obtained were 8.4 mg/L and 7.0 mg/L for MT4 and MT3 strains, respectively [5].

*Sulfolobus solfataricus *and the closely related hyperthermophilic crenarchaeota *Sulfolobus acidocaldarius* and *S. tokodaii* were recently studied by Koerdt et al. [46] for biofilm formation. Biofilm analysis by confocal laser scanning microscopy demonstrated that these three strains form very different communities ranging from simple carpet-like structures in *S. solfataricus* to high-density tower-like structures in *S. acidocaldarius* in static systems. Moreover, lectin staining indicated that all three strains produced extracellular polysaccharides containing glucose, galactose, mannose, and N-acetylglucosamine once biofilm formation was initiated [46].

Another example of exopolysaccharide as constituent of archaea biofilm is produced by *Archaeoglobus fulgidus*, the best characterized *Archaeoglobus *species. *A. fulgidus* is an anaerobic marine hyperthermophile that obtains energy by dissimilatory sulfate reduction by using H_2_, lactate, or pyruvate as the electron donor. The ability of *A. fulgidus *to colonize widely separated areas successfully suggests that it has evolved mechanisms for surviving fluctuations in temperature, concentrations of nutrients, and potentially toxic compounds [6]. These archaea were found to form a biofilm in response to environmental stresses. The biofilm is an heterogeneous, morphologically variable structure containing proteins, polysaccharides, and metals. The production of the biofilm can be induced by nonphysiological extremes of pH and temperature, by high concentrations of metals, and by addition of antibiotics, xenobiotics, or oxygen. Lapaglia and Hartzell [6] demonstrated that cells within the biofilm showed an increased tolerance to otherwise toxic environmental conditions. Moreover, metals sequestered within the biofilm stimulated the growth of *A. fulgidus *cells in a metal-depleted medium suggesting that cells may produce biofilm as a mechanism for concentrating cells and attaching to surfaces, as a protective barrier, and as a reserve nutrient. Since similar biofilms are formed by *Archaeoglobus profundus*, *Methanococcus jannaschii*, and *Methanobacterium thermoautotrophicum*, the biofilm formation might be a common stress response mechanism among the Archaea [8].

A soluble exopolysaccharide produced by *Thermococcus litoralis*, apparently involved in the formation of a biofilm, was studied by Rinker and Kelly [7]. Analysis of the acid-hydrolyzed exopolysaccharide yielded mannose as the only monosaccharidic constituent. More recently, Rinker and Kelly [40] studying the effect of carbon and nitrogen sources on growth dynamics and exopolysaccharide production for the archaeon *Thermococcus litoralis *and the bacterium *Thermotoga maritima*, found that not only *T. litoralis* was unable to utilize NH_4_Cl as a nitrogen source, but its growth was even inhibited at certain levels. Moreover, exopolysaccharide production for both organisms was significant and increased with increasing dilution rate. In particular, *T. litoralis *produced more than twice as much total EPS as *T. maritima *under optimal growth conditions (~0.32 g EPS/L and ~0.1 g EPS/L at dilution rate 0.4 h^−1^ for *T. litoralis* and *T. maritima*, resp.). In addition, in the presence of 1 g/L NH_4_Cl, the EPS/CDW by *T. litoralis* was found to increase significantly with increasing dilution rate (~10 EPS/CDW at dilution rate 0.7 h^−1^ in the presence of 1 g/L NH_4_Cl versus ~2 EPS/CDW at the same dilution rate but in absence of NH_4_Cl) [40].

## 5. EPS Productions and Biotechnological Applications

Although both the composition and the amount of EPS produced by a microorganisms are genetically determined traits, they are highly influenced by media components and cultivation conditions. EPSs synthesis is generally favored by presence of carbon source in excess, concomitant with limitation by another nutrient (e.g., nitrogen, oxygen) [15]. Fermentation is an extremely versatile process technology for producing value-added products such as microbial biopolymers. Particularly, microbial polysaccharide production is greatly influenced by fermentation conditions. In fact the structure, composition, and viscosity of EPSs depend on several factors, such as the composition of the culture medium, carbon and nitrogen sources and precursor molecules, mineral salts, trace elements, type of strain, and fermentation conditions such as pH, temperature, oxygen concentration, and agitation [15]. Microorganisms used as industrial or technical producers of extracellular polysaccharides are mainly pathogenic bacteria. Species of *Xanthomonas*, *Leuconostoc*, *Pseudomonas, *and *Alcaligenes *which produce xanthan, dextran, gellan, and curdlan, respectively, are the most well known and most industrially used. Actually, the EPSs produced by lactic acid bacteria (LAB), which are already accepted as GRAS (generally recognised as safe) represent the most suitable polymers for the food industry. They are widely employed in the dairy industry since the *in situ *production of their EPSs improves the texture of fermented dairy products and also confers health benefits as a result of their immunostimulatory, antitumoral or cholesterol-lowering activity [47].

Dextran (produced by LAB such as *Leuconostoc mesenteroides *and the mesophilic dental pathogen *Streptococcus mutans*), xanthan gum (the EPS from the plant pathogen *Xanthomonas campestris *pv. *campestris *bacterium), gellan (produced by the nonpathogenic bacterium *Pseudomonas elodea*), and curdlan (produced by the alkaline tolerant mesophilic pathogen *Alcaligenes faecalis*) are some examples of commercial microbial polysaccharides that entered the market. Because of the pathogenicity of the commercial EPS-producing strains, in recent years significant progress has been made in discovering and developing novel and functional EPSs from extremophilic producer strains [15]. 

Currently, despite the vast number and biodiversity of the extremophilic producers of EPS, these non-toxic and biodegradable polymers represent only a small fraction of the current polymer market. These few marketable exopolysaccharides derived from extremophiles belong only to the bacteria domain: actually no EPS produced by Archaea has a commercial application. The high production costs and the poor physicochemical properties (if compared with those of industrial EPSs from plant such as guar gum, cellulose, pectin and starch, and from seaweed as alginate and carrageenan), make the microbial EPSs not suitable for profit purpose [48, 49]. The fermentation media that can represent almost 30% of the cost for a microbial fermentation usually are made of expensive nutrients such as yeast extract, peptone, and salts. In order to maximize the cost-effectiveness of the process, recent works shifted to using multicomponent feedstock systems, and the synthetic media were replaced by cheaper alternatives: molasses were successfully used for fermentative production of commercial polysaccharides such as curdlan [50], xanthan [51], dextran [52], and gellan [53], and the use of spent malt grains, apple pomace, grape pomace and citrus peels for xanthan production by solid state fermentation [54], the use of olive mill wastewater in xanthan production [55] are some examples.

Besides the use of cheaper substrates, the reduction of production costs may involve the improvement of product yields by optimizing fermentation conditions or developing higher yielding strains (e.g., by mutagenesis or genetic manipulation), and by optimizing downstream processing. Moreover, the interest for the development of microbial EPSs could be related to their use in high-value market niches, such as, cosmetics, pharmaceuticals and biomedicine, where traditional polymers fail to comply with the required degree of purity or lack some specific functional properties. In these high-value applications, quality and purity products wholly surpass the cost production and product yield issues, identifying in these interesting biopolymers suitable candidates for biotechnological applications [56].

## 6. PHAs: Characteristics and Compositions

Microorganisms are capable of forming a variety of intracellular and spherical inclusions. These inclusions can be surrounded by a phospholipid membrane and divided into inorganic inclusions, such as for example, magnetosomes (iron oxide core), and organic inclusions such as for example, biopolyester (PHAs: polyhydroxyalkanoates) granules (polyester core) [27]. Polyhydroxyalkanoates are polyesters composed of hydroxy fatty acids, which represent a complex class of storage polyesters. Poly-*β*-hydroxybutyrate (PHB) is the best known polyhydroxyalkanoate. PHAs are deposited as water-insoluble cytoplasmic nanosized inclusions. These spherical shell-core particles are composed of a polyester core surrounded by phospholipids and proteins; they crystallize after solvent extraction and exhibit rather high molecular weights (ranging from about 5 × 10^5^ to 5 × 10^6^ Da), thermoplastic and elastomeric properties, and some other interesting physical and material properties. PHAs are synthesized by several microorganisms as reserves of carbon and energy in the presence of excess of a source of carbon and usually when an essential nutrient restricts the cellular growth. It is now well recognized that these lipid inclusions are accumulated by many bacteria as they enter the stationary phase of growth to be used later as an internal reserve [19]. When carbon and energy are required, PHA is normally depolymerised to D(−)-hydroxybutyric acid and then metabolized to acetoacetate and acetoacetyl-CoA. The diversity of different monomers that can be incorporated into PHAs, combined with biological polymerization systems that generate high-molecular weight materials, has led to an enormous range of new potentially available polymers. Besides the typical properties described above, an important characteristic of PHAs is their biodegradability. 

PHAs are found in a wide range of different Gram-positive and Gram-negative bacteria, as well as in some haloarchaeal species (Table 2) belonging to the genera *Haloferax*, *Haloarcula*, *Natrialba*, *Haloterrigena*, *Halococcus*, *Haloquadratum, Halorubrum*, *Natronobacterium*, *Natronococcus *and *Halobacterium. *These latter are capable of synthesizing short-chain-length polyhydroxyalkanoates (SCL-PHAs), PHB and copolymers of poly-*β*-hydroxybutyrate-co-3-hydroxyvalerate (PHBV) as carbon and energy storage [12, 20–29]. Notably, the presence of PHA in the cells has also been used as a chemotaxonomic marker to help in the identification of a new isolate [12]. Among the halophilic archaea, *Haloferax mediterranei* is one of the best-studied strains due to its capability to accumulate large amounts of copolymers of PHBV, and therefore it has been evaluated as one of the most promising candidate prokaryotes for the industrial production of PHBV [63, 64]. The PHA accumulated by *H. mediterranei *was originally reported to be PHB [26, 57] but recently it has been reevaluated as PHBV [64, 65]. Also *Haloarcula hispanica *(previously deposited as *Halobacterium hispanicum*) was originally indicated as PHB producer [57] but subsequent studies reported a PHBV production [58].

The presence of PHA granules in haloarchaea was first reported by Kirk and Ginzburg [66]. The strain was called at that time “*Halobacterium* sp. from the Dead Sea,” but later it was identified as *Haloarcula marismortui* [67]. Chemically, PHAs consist mostly of PHB and copolymers of PHBV. 

More than 100 different monomer units have been found as constituents of PHAs in various microorganisms depending upon the carbon source supplemented to the culture medium [68]. The monomers of PHAs frequently found are the 3-hydroxyalkanoates (3-HAs) of 3–14 carbon atoms and/or 4-HAs and 5-HAs of three to five carbon atoms, which may be saturated or unsaturated and straight or branched chains containing aliphatic or aromatic side groups [68].

Among the several biodegradable polymers under development, PHAs have attracted much attention because of the similarity of their properties to those of the conventional petrochemical-derived plastics and their complete biodegradability in various environments. The film type PHAs show gas-barrier properties comparable to those of poly(vinyl chloride) and poly(ethylene terephthalate); therefore, PHAs can compete with nondegradable polymer used in the packaging industry and, in the same time, represent the ideal candidate to satisfy the increasing demand for environmentally compatible materials derived from renewable resources [69, 70]. PHAs of different chemical structures are under investigation for their potential applications in controlled drug release, sutures, bone plates, wound dressing, paramedical disposables, and therapeutic devices [71–74]. Moreover, biodegradability, thermoplastic properties, and biocompatibility make these materials suitable for several applications in packaging industry, medicine, pharmacy, agriculture, and food industry or as raw materials for the synthesis of enantiomerically pure chemicals and the production of paints [75].

Several methods have been described and used to collect the biopolymer and define its content in bacteria. All of them require time-consuming and difficult procedures, large use of organic solvent, several purification steps, dispersion strategy of sodium hypochlorite and chloroform, and enzyme digestions [76, 77]. The methods described are not profitable due to the difficult and slow processes, which result in a low bioproducts recovery yield and high environmental impact. Recently, Strazzullo et al. [78] proposed a simplified and rapid extraction method in which solvents were not used. In this simple methodology sodium dodecyl sulphate was directly added to dispersed biomass of culture microorganisms in distilled water followed by shaking, heat treatment, and washing steps. Subsequently, mass spectroscopy (MS) and ^1^H-^13^C NMR analysis were used to chemically characterize the biopolymer.

## 7. Polyester Synthase: The Key Enzyme of PHA Biosynthesis

Polyester synthases are the key enzymes of polyester biosynthesis and catalyse the conversion of (*R*)-3-hydroxyacyl-CoA thioesters to polyesters with the concomitant release of CoA [79]. 

Microorganisms transform sugars and fatty acids to PHAs through three different metabolic pathways, which involve as intermediate either acetyl-CoA or acyl-CoA and end with monomer polymerization by PHA synthases [80]. In general PHB synthesis starts from acetyl-CoA and proceeds via generation of acetoacetyl CoA and 3-hydroxybutyryl-CoA. Initially condensation of two acetyl-CoA molecules takes place to form acetoacetyl-CoA, in a reaction that is catalyzed by *β*-ketothiolase (PhaA). Reduction of acetoacetyl-CoA is carried out by an NADPH-dependent acetoacetyl-CoA dehydrogenase (PhaB). Lastly, the (R)-3-hydroxybutyryl-CoA monomers are polymerized into P(3HB) by P(3HB) polymerase (PhaC) [79, 81]. The genes and enzymes involved in the synthesis of PHAs have evolved features peculiar of different microbial groups. The ability of microorganisms to synthesize a particular form of PHA is mainly due to the substrate specificity of PHA synthases. Over the past few decades, extensive research has been devoted to the study of PHA synthases in the domain of bacteria where these enzymes may be divided into four classes according to their substrate specificity and subunit composition [79]. PHA synthases belonging to class I utilize CoA thioesters of 3-HAs, 4-HAs, and 5-HAs comprising three to five carbon atoms whereas class II polymerases direct their specificity towards CoA thioesters of 3-HAs with six to fourteen carbon atoms, and of 4-HAs and 5-HAs. Synthases of both classes I and II are encoded by *phaC* gene. The typical bacterial type III synthase is composed of two subunits named PhaE and PhaC with similar molecular weight (about 40 kDa) that possess substrate specificities similar to class I, although the PhaCE subunit can also polymerize 3-HAs with six to eight carbon atoms. Class IV synthases resemble the class III PHA synthases, but PhaE is replaced by PhaR (molecular mass of approx. 20 kDa): they are coded by genes (*phaC* and *phaR*) that utilize 3-HA monomers with three to five carbon atoms [79]. While the metabolic pathways of PHAs in bacteria have been characterized in detail, the genes involved in PHA biosynthesis in haloarchaea were not recognized until recently, when the first archaeal-type *phaEC *genes encoding a putative class III PHA synthase were identified and characterized in *Haloarcula marismortui*, *Haloarcula hispanica,* and *Haloferax mediterranei* [21, 58, 82]. The archaeal PHA synthases present in these species are composed of two subunits, PhaE and PhaC that are homologous to the class III PHA synthases from bacteria, showing a longer C-terminal extension in the PhaC subunit (c.a. 1430 bp) and the presence of conserved residues (e.g., the Cys-Asp-His catalytic triad). In contrast, the PhaE (c.a. 550 bp) subunit is much smaller than its bacterial counterpart and lacks hydrophobic and amphiphilic amino acids for granule association instead present in the corresponding bacterial class III enzymes [21, 58]. Lu et al. [58] for the first time cloned the gene cluster (*phaEC*_Hme_) encoding a PHA synthase in *Haloferax mediterranei*, showing that both the PhaE_Hme_ and the PhaC_Hme_ proteins were strongly bound to the copolymer PHBV granules. It is noteworthy that as in bacteria, the PHA synthase-encoding genes (*phaEC*_Hme_) in *Haloferax mediterranei* are clustered and cotranscribed, and that both the *PhaC*_Hme_ and the *PhaE*_Hme_ protein subunits are indispensable for the PHBV synthesis from multiple unrelated carbon sources. Moreover, the authors reported that the knockout of the *phaEC*_Hme_ genes in *Haloferax mediterranei *led to a complete loss of PHBV synthesis, and only a complementation with the whole *phaEC*_Hme_ genes could restore to this mutant the capability for PHBV accumulation.

A recent review [70] listed the PHA production by halophilic Archaea and Bacteria and underlined, through the multiple alignment of amino acids sequence of phaC subunit, how the primary structure of PHA synthases of haloarchaea studied (*Haloferax mediterranei*,* Halogeometricum borinquense*,* Haloquadratum walsbyi*, *Halorhabdus utahensis*,* Haloarcula marismortui*,* Haloarcula hispanica, and Halorhodospira halophila*) shared very high identities. The phylogenetic tree reported by Quillaguamán et al. [70] based on the phaC synthases in halophilic and nonhalophilic microorganisms belonging to both bacteria and archaea domains revealed that some archaea and bacteria that share the closest genetic affiliations among PHA polymerases proliferate in similar habitats of marine origin. Phylogenetic tree based on the PhaC and PhaE/R subunits from some representative bacteria and haloarchaea further suggested that the PHA synthase from haloarchaea belongs to a novel subgroup of the class III family [58] and finally indicated that PHA biosynthesis genes in haloarchaea might have been acquired from bacteria through horizontal gene transfer [81]. Anyway, not all the 12 *Halobacteriaceae* species (*Haloarcula marismortui* ATCC 43049, *Halobacterium salinarum* NRC-1 ATCC700922, *Halobacterium salinarum* R1, *Haloferax volcanii* DS2, *Halogeometricum borinquense* DSM 11551, *Halomicrobium mukohataei* DSM 12286, *Haloquadratum walsbyi* DSM 16790, *Halorhabdus utahensis* DSM 12940, *Halorubrum lacusprofundi* ATCC 49239, *Haloterrigena turkmenica* DSM 5511, *Natrialba magadii* ATCC 43099, *Natronomonas pharaonis* DSM 2160) for which the whole-genome sequences are available and contain both the *phaC* and the *phaE* genes [25]. In particular, the species *Halogeometricum borinquense*, *Halomicrobium mukohataei*, *Halorhabdus utahensis*, *Haloquadratum walsbyi* and *Haloterrigena turkmenica* contain only the *phaC *gene [25] in addition to the already cited *Haloarcula hispanica, Haloarcula marismortui* and *Haloferax mediterranei* that present the *phaEC *genes. Moreover, *Hbt. salinarum* NRC-1 ATCC700922 contains homologues of *phaA* and *phaB* genes, but not of *phaC* [81].

Characterization of PHA synthases in halophilic archaea began with the PHB synthase of strain 56 [83]; this strain has been classified recently as *Halopiger aswanensis* [62]. This enzyme, like some other enzymes produced by members of the family *Halobacteriaceae*, shows a high thermostability (up to 60°C) and was found to be mainly granule associated. Hezayen et al. [83] reported that PHA synthase is covalently linked to the PHB core of the granule by a thioester bond. Generally, enzymes from extremely halophilic archaebacteria require high salt concentrations for their biological activity and stability. High salt concentrations are required to compensate the high protein surface charge of these enzymes [84]. Accordingly, the soluble PHB synthase did not exhibit enzyme activity in the absence of salts and possesses a very narrow substrate specificity. Notably *H. aswanensis* synthesizes only PHB even in the presence of other hydroxyalkanoate monomers, for example, 3-HV, 4-HB, in the culture medium [83].

## 8. PHA Productions

One of the major drawbacks of employing PHA in a wide range of applications is its high production cost. Consequently, much effort has been devoted to reduce its production cost by improving bacterial strains, efficient fermentation and recovery processes [86]. Currently, PHAs are industrially produced by the company Metabolix in USA under the commercial name Mirel using a recombinant *Escherichia coli* strain [87, 88].

In general haloarchaea represent the ideal candidate for PHA production. *Haloferax mediterranei* was used to produce PHA under a hypersaline condition in which very few organisms can survive. The extreme conditions of salinity in which these organisms grow are useful to avoid the contamination problem reducing the sterility requirements and the production costs [26, 57]. In addition, it is relatively easy to recover PHA pellet from haloarchaea, compared to other PHA accumulating microorganisms, in that they can be easily lysed in the distilled water and release the PHA pellet that can be recovered by low speed centrifugation [57]. Lillo and Rodriguez-Valera [26] reported the effects of culture conditions for PHA production in *Haloferax mediterranei*. They found that PHA accumulation starts during the logarithmic phase, increases with the biomass and reaches a peak at the beginning of the stationary phase. PHA synthesis is delayed with respect to biomass development, reaching a maximum rate of synthesis at the end of the exponential phase. Moreover, the authors found that phosphate limitation is essential for PHA accumulation in large quantities and that glucose and starch are the best carbon sources reaching a production of ca. 6 g of PHA per liter in batch culture, being 60% of the total biomass dry weight. Subsequently, studies conducted by Don et al. [65] established the chemical structure of the PHA produced by *H. mediterranei *as the copolymer PHBV containing 10.7% of 3-HV and indicated that the fed-batch fermentation using glucose as carbon source gave a maximum PHA content of 48.6 wt.% and a volumetric production of PHA of 0.36 g^−1^ L^−1^ h^−1^. 

Starting from the concept that the cost of carbon source is critical for reducing the production cost of PHA production [86, 89, 90], recently many authors discussed frequently in the literature a cheaper way to obtain an economically competitive PHA production from renewable resources (Table 3). The use of waste products as substrates for extremophilic biomass production is an attractive option for producing metabolites for commercial exploitation. A study carried out by Huang et al. [60] demonstrated that different low cost raw materials can be used as carbon source in order to reduce the production cost of PHA by *H. mediterranei*. The materials used were the extruded rice bran and the extruded corn starch got by the extruder machine using the native agricultural wastes previously treated with alpha-amylase. The authors, by employing pH-stat control strategy to maintain pH at 6.9–7.1 in a 5-liter jar bioreactor, using a combination of extruded rice bran extruded cornstarch (1 : 8, w/w), obtained a cell concentration of 140 gL^−1^, and a PHA concentration of 77.8 gL^−1^, a PHA content of 55.6 wt.% and the productivity was increased to 0.71 g^−1^ L^−1^ h^−1^ [60]. Koller et al. [63] pointed out the attention on whey, the major byproduct from cheese and casein production, and proposed it as a feed stock for the biotechnological production of PHA by *H. mediterranei*. The authors, highlighting how this cheap raw material, rich of lactose, constitutes a surplus product for the dairy industry, suggested a solution to waste problem combined with the development of a bio-inspired technological process [63, 64, 91]. Moreover, they found that using hydrolyzed whey, *H. mediterranei* produced the copolymer PHBV containing 6.0% of 3-HV, the PHA content was 72.8 wt.% but a low volumetric production of PHA was recorded (0.09 g^−1^ L^−1^ h^−1^). Further, a PHA terpolyester, P(3HB-*co*-21.8% 3HV-*co*-5.1% 4HB) with an increased 3HV fraction as well as 4-hydroxybutyrate (4HB) building blocks, was accumulated by feeding of hydrolyzed whey sugars plus sodium valerate and gamma butyrolactone [64]. In this latter case, the PHA productivity and the PHA yield were 0.14 g^−1^ L^−1^ h^−1^ and 87.5 wt.%, respectively, even if the molecular weight decreased from 1,047 kDa, using the only hydrolysed whey, to 987 kDa [64]. Overall these studies, *H. mediterranei* demonstrates a good capability to synthesize polymeric materials that can be tailored for different applications [70].

Taran and Amirkhani [59] investigated the optimization of PHB production by *Haloarcula* sp. IRU1, an halophilic archaea isolated from hypersaline Urmia lake in Iran. Various experiments in a batch culture system were carried out at different glucose, phosphorus, and nitrogen concentrations and at different temperatures. By these experiments, optimum production conditions were determined using the Taguchi method, a good option for optimization of biotechnological processes for microbial synthesis [92]. They found that the highest PHB production by *Haloarcula* sp. IRU1 (63.0% of cell dry weight) was achieved in the presence of 2 g/L glucose, 0.2 g/L NH_4_Cl, and 0.004 g/L KH_2_PO_4_ at 42°C. Recently, Taran [85] described the ability of *Haloarcula* sp. IRU1 to produce poly(3-hydroxybutyrate) by the utilization of petrochemical wastewater. The wastewater of some petrochemical plants, in addition to hydrocarbons, is rich in chlorinated chemicals that can be degraded by numerous microorganisms including bacteria and fungi [85]. The author demonstrated the effectiveness and feasibility of *Haloarcula* sp. IRU1 for biodegradation of petrochemical waste water: using these wastes as sole carbon source at 2% (v/v) in the growth media, this archaeon produced up to 46% PHB of cell dry weight suggesting an ecocompatible and cheaper production of poly(3-hydroxybutyrate) [85].

Recently, Di Donato et al. [61] studied the reuse of industrial vegetable wastes as growth media for extremophile biomass fermentation thus providing a cheaper way to produce biotechnological extremozymes or biopolymers using zero-cost feedstocks. One of the microorganisms employed, the haloarchaeon *Haloterrigena hispanica* strain FP1, accumulates intracellular PHB previously identified as poly(3-hydroxybutyrate) when grown on complex standard medium [28]. The authors found that FP1 cells grown on carrot wastes as sole carbon were able to produce a comparable amount of PHB (0.13% of CDW) with respect to that produced when the growth was carried out on complex standard media (0.14% of CDW) thus suggesting an alternative and low environmental impacting method for vegetable wastes management. 

Since fermenters must be built of materials resistant to corrosion by the media required for growth of halophiles, a novel corrosion-resistant bioreactor composed of polyetherether ketone, tech glass (borosilicate glass), silicium nitrate ceramics, and silicon nitrite ceramics was used for the cultivation of two extreme halophilic archaea isolates that produce poly-*gamma*-glutamic acid and poly-*beta*-hydroxybutyric acid, respectively [22]. Batch fermentations on *n*-butyric acid as carbon source yielded a cell density (dry biomass) of 2.3 g L^−1^, with the accumulation of poly-*beta*-hydroxybutyric acid comprising up to 53% of the dry biomass.

Nicolaus et al. [12] reported the study of obligate halophilic archaea from an unexplored site in Tunisia (Monastir). The three strains (T5, T6, andT7), although different in colony and cell morphology, were similar in a homopolyester synthesis, identified spectroscopically (^1^H and ^13^C-NMR) as polyhydroxybutyrate. Whereas the three Tunisian isolates biosynthesized the same polyester, they were found to produce exopolysaccharides (see the Section 7) with different chemical structures. The isolates were able to synthesize PHB only if they were grown on minimal medium containing glucose: in fact PHB was not detected when the strains were grown in the standard complex medium. In particular, the polyester concentration of T5 strain was 0.5% of dried cells weight when the strain was grown in the minimal medium with glucose; moreover the yield of the polymer reached its maximum in the presence of molasses (1.0% of dried cells weight).

## Figures and Tables

**Table 1:** EPSs produced by archaeal species.

Microorganism	Carbon source	Chemical composition	Yield (w/v)	Reference
*Haloferax mediterranei*	Glucose	→4)-*β*-D-Glc*p*NAcA-(1→6)-*α*-D-Man*p*-(1→4)-*β*-D-Glc*p*NAcA-3-O-SO_3_^−^-(1→	3.0 (g L^−1^)	[11, 14]
*Haloferax denitrificans*	Glucose	→4)-*β*-D-Glc*p*A2,3NAc-(1→4)-*β*-D-Glc*p*A2,3NAc-(1→4)-*α*-D-Glc*p*A2,3NAc-(1→3)-*α*-D-Gal*p*-(1→	n.d.	[39]
*Haloferax gibbonsii*	Glucose	→4)-*β*-D-Man*p*-(1→4)-*β*-D-Man*p*-(1→4)-*α*-D-Gal*p*-(1→3)-*β*-D-Gal*p*-(1→	n.d	[13]
		3 2		
		↑ ↑		
		1 1		
		*α*-D-Gal*p*-(1→2)- *α*-L-Rha*p* *α*-D-Gal*p *		
*Haloarcula japonica* strain T5	Glucose	Man/Gal/GlcA 2 : 1 : 3	370 (mg L^−1^)	[12]
*Haloarcula *sp. strain T6	Glucose	Man/Gal/Glc 1 : 0.2 : 0.2	45 (mg L^−1^)	[12]
*Haloarcula* sp. strain T7	Glucose	Man/Gal/Glc 1 : 0.2 : 0.2	35 (mg L^−1^)	[12]
*Sulfolobus solfataricus * strain MT4	Glucose	Glc/Man/GlcN/Gal 1.2 : 1.0 : 0.18 : 0.13	8.4 (mg L^−1^)	[5]
*S. solfataricus * strain MT3	Glucose	Glc/Man/GlcN/Gal 1.2 : 1.0 : 0.77 : 0.73	7.0 (mg L^−1^)	[5]
*Thermococcus* *litoralis *	Maltose	Man	0.33 (g L^−1^)	[7, 40]

Chemical composition was determined by NMR spectroscopy analysis or by HPAE-PAD analysis after acid hydrolysis. n.d.: not determined; Gal: galactose; Glc: glucose; GlcA: glucuronic acid; GlcN: glucosamine; GlcANAc: N-acetylglucosamine; GlcNAcA: 2-acetamido-2-deoxyglucuronic acid; Glc*p*A2,3NAc: 2,3-diacetamido-2,3-dideoxy-D-glucopyranosiduronic acid; Man: mannose; Rha: rhamnose.

**Table 2:** PHAs produced by archaeal species.

Microorganism	Carbon source	Type	Yield	Reference
*Haloarcula hispanica* (previously deposited as *Halobacterium hispanicum*)	Yeast extract/Glc	PHB	2.4 (% w/w of CDW)	[57]
*H. hispanica*	Yeast extract/Glc	PHBV	0.58 ± 0.03 (g L^−1^)	[58]
*Haloarcula* *marismortui *	Yeast extract/Glc	PHB	21 (% w/w of CDW)	[21]
*Haloarcula *sp. IRU1	Glc	PHB	63 (% w/w of CDW)	[59]
*Haloarcula japonica* (strain T5)	Glc	PHB	0.5 (% w/w of CDW)	[12]
*Halobacterium noricense*	Yeast extract/triptone	PHB/PHBV	0.08/0.03 (% w/w of CDW)	[25]
*Halobiforma haloterrestris* (strain 135^T^)	Yeast extract/butyric acid	PHB	40 (% w/w of CDW)	[23]
*H. haloterrestris* (strain 135^T^)	Yeast extract/casamino acids or proteose peptone	PHB	15 (% w/w of CDW)	[23]
*Halococcus* *dombrowskii *	Yeast extract/HyCase	PHB/PHBV	0.15/0.01 (% w/w of CDW)	[25]
*Halococcus* *salifodinae *	Yeast extract/HyCase	PHB/PHBV	0.05/0.01 (% w/w of CDW)	[25]
*Haloferax mediterranei*	Yeast extract/Glc	PHB	17 (% w/w of CDW)	[57]
*H. mediterranei*	Glc	PHBV	23 (g L^−1^)	[60]
*H. mediterranei*	Starch	PHB	6.48 (g L^−1^)	[26]
*H. mediterranei*	Bacto Casamino Acids/yeast extract	PHBV	1.33 ± 0.05 (g L^−1^)	[58]
*H. mediterranei*	Yeast extract/starch	PHBV	1.74 ± 0.04 (g L^−1^)	[58]
*Haloferax gibbonsii*	Yeast extract/Glc	PHB	1.2 (% w/w of CDW)	[57]
*Haloferax volcanii*	Yeast extract/Glc	PHB	7 (% w/w of CDW)	[57]
*Haloterrigena hispanica*	Yeast extract/casamino acids	PHB	0.14 (% w/w of CDW)	[28, 61]
*Halopiger aswanensis* (strain 56)	Yeast extract/sodium acetate/butyric acid	PHB	34 (% w/w of CDW)	[22, 62]
*Natronobacterium gregoryi *	Yeast extract/casamino acids	PHB/PHBV	0.1/0.03 (% w/w of CDW)	[25]

Abbreviations: CDW: cell dry weight; Glc: glucose; PHB: poly (3-hydroxybutyrate); PHBV: poly (3-hydroxybutyrate-*co*-3-hydroxyvalerate).

**Table 3:** PHAs produced by archaeal species grown on waste sources.

Microorganism	Carbon source	Type	Yield	Reference
*Haloferax mediterranei*	ECS	PHBV	24.2 (g L^−1^)	[60]
*H. mediterranei*	ERB/ECS	PHBV	77.8 (g L^−1^)	[60]
*H. mediterranei*	EWB/ECS	PHBV	52.7 (g L^−1^)	[60]
*H. mediterranei*	NWB/ECS	PHBV	28.0 (g L^−1^)	[60]
*H. mediterranei*	Hydrolyzed whey	PHBV	5.5 (g L^−1^)	[63]
*H. mediterranei*	Hydrolyzed whey	PHBV	12.2 (g L^−1^)	[64]
*H. mediterranei*	Hydrolyzed whey	PHBVB	14.7 (g L^−1^)	[64]
*Haloarcula *sp. IRU1	Petrochemical waste water	PHB	46 (% w/w of CDW)	[85]
*Haloarcula japonica *(strain T5)	Molasses	PHB	1.0 (% w/w of CDW)	[12]
*Haloterrigena hispanica*	Carrot waste	PHB	0.13 (% w/w of CDW)	[61]

CDW: cell dry weight; PHB: poly(3-hydroxybutyrate); PHBV: poly(3-hydroxybutyrate-*co*-3-hydroxyvalerate); PHBVB: poly(3-hydroxybutyrate-*co*-3-hydroxyvalerate-*co*-4-hydroxybutyrate); ECS: extruded cornstarch; ERB: extruded rice bran; EWB: extruded wheat bran; NWB: native wheat bran.
